# Supplementary material for: Bundle sheath suberisation is required for C4 photosynthesis in a Setaria viridis mutant
Source: Commun Biol. 2021 Feb 26;4:254. doi: 10.1038/s42003-021-01772-4 (PMC7910553; doi:10.1038/s42003-021-01772-4)
Supplement: Supplementary file 3 — Description of Additional Supplementary Files [file 42003_2021_1772_MOESM3_ESM.pdf]

Description of Additional Supplementary Files

File name: Supplementary Data 1

Description: Raw data values obtained from the measurements performed in this study.

File name: Supplementary Video 1

Description: Z-stack of Setaria viridis wildtype leaf after clearing and staining with Nile Red and Calcofluor White.

File name: Supplementary Video 2

Description: Z-stack of Setaria viridis mutant leaf after clearing and staining with Nile Red and Calcofluor White.
