# Supplementary material for: Bundle sheath suberisation is required for C4 photosynthesis in a Setaria viridis mutant
Source: Commun Biol. 2021 Feb 26;4:254. doi: 10.1038/s42003-021-01772-4 (PMC7910553; doi:10.1038/s42003-021-01772-4)
Supplement: Supplementary file 7 — Reporting Summary [file 42003_2021_1772_MOESM7_ESM.pdf]

nature research

Corresponding author(s): Florence Danila

Last updated by author(s): Jan 18, 2021

Reporting Summary

Nature Research wishes to improve the reproducibility of the work that we publish. This form provides structure for consistency and transparency in reporting. For further information on Nature Research policies, see Authors & Referees and the Editorial Policy Checklist .

Statistics

For all statistical analyses, confirm that the following items are present in the figure legend, table legend, main text, or Methods section.

n/a	Confirmed	
☐	☒	The exact sample size (*n*) for each experimental group/condition, given as a discrete number and unit of measurement
☐	☒	A statement on whether measurements were taken from distinct samples or whether the same sample was measured repeatedly
☐	☒	The statistical test(s) used AND whether they are one- or two-sided<br>*Only common tests should be described solely by name; describe more complex techniques in the Methods section.*
☐	☒	A description of all covariates tested
☐	☒	A description of any assumptions or corrections, such as tests of normality and adjustment for multiple comparisons
☐	☒	A full description of the statistical parameters including central tendency (e.g. means) or other basic estimates (e.g. regression coefficient) AND variation (e.g. standard deviation) or associated estimates of uncertainty (e.g. confidence intervals)
☐	☒	For null hypothesis testing, the test statistic (e.g. *F*, *t*, *r*) with confidence intervals, effect sizes, degrees of freedom and *P* value noted<br>*Give P values as exact values whenever suitable.*
☐	☒	For Bayesian analysis, information on the choice of priors and Markov chain Monte Carlo settings
☐	☒	For hierarchical and complex designs, identification of the appropriate level for tests and full reporting of outcomes
☐	☒	Estimates of effect sizes (e.g. Cohen's *d*, Pearson's *r*), indicating how they were calculated

Our web collection on statistics for biologists contains articles on many of the points above.

Software and code

Policy information about availability of computer code

Data collection	Leica SP8 confocal microscope with LASX software and Hitachi 7100 transmission electron microscope were used to collect images.<br>All bioinformatics analysis-related softwares used in this study are listed in the Supplementary information of this manuscript.
Data analysis	LASX software (Leica Microsystems), which comes with the Leica SP8 confocal microscope unit was used to process (i.e. merging multiple channels, maximum image projection) all confocal images.<br>Fiji software was used for light and electron microscopy-related measurements.<br>All gas chromatography/mass spectrophotometry data processing softwares used in this study are stated in the Methods section of this manuscript.<br>All bioinformatics analysis-related softwares used in this study are stated in the Supplementary information of this manuscript.<br>OriginPro 9.1 (OriginLab Corporation) was used for generating plots and statistical analyses.

For manuscripts utilizing custom algorithms or software that are central to the research but not yet described in published literature, software must be made available to editors/reviewers. We strongly encourage code deposition in a community repository (e.g. GitHub). See the Nature Research guidelines for submitting code & software for further information.

Data

Policy information about availability of data

All manuscripts must include a data availability statement. This statement should provide the following information, where applicable:

- Accession codes, unique identifiers, or web links for publicly available datasets
- A list of figures that have associated raw data
- A description of any restrictions on data availability

The authors declare that the data supporting the findings of this study are available within the paper and its supplementary information files. The whole genome sequencing data generated for this study has been uploaded to NCBI's Short Read Archive database under BioProject ID PRJNA692561. Metabolomics data generated from this study have been deposited to the EMBL-EBI MetaboLights database (DOI: 10.1093/nar/gkz1019, PMID:31691833) with the identifier

MTBLS2380. The complete dataset can be accessed here https://www.ebi.ac.uk/metabolights/MTBLS2380.

Field-specific reporting

Please select the one below that is the best fit for your research. If you are not sure, read the appropriate sections before making your selection.

- [x] Life sciences
- [ ] Behavioural & social sciences
- [ ] Ecological, evolutionary & environmental sciences

For a reference copy of the document with all sections, see nature.com/documents/nr-reporting-summary-flat.pdf

Life sciences study design

All studies must disclose on these points even when the disclosure is negative.

Sample size: A minimum sample size of 4 was used as the plants (mutant and wildtype control) used for this study were grown under one controlled environment.

Data exclusions: No data were excluded from the analysis.

Replication: Plants (mutant and wildtype control) were grown at least three times within the period of March to August 2019 to allow measurements of the different parameters described in this study. We grew the mutant plants from the homozygous M5 seed stock and we made sure that we use the same plant developmental stage as described in the Methods. Each time the measured values were replicated.

Randomization: Allocation was not random. Plants used for this study were categorised as mutant and wildtype control. The mutant was obtained from careful screening of large mutant population, where a distinct phenotype was associated to the mutant as a result of the mutation.

Blinding: Blinding was not possible as the mutant displays a distinct phenotype compared to wildtype control.

Reporting for specific materials, systems and methods

We require information from authors about some types of materials, experimental systems and methods used in many studies. Here, indicate whether each material, system or method listed is relevant to your study. If you are not sure if a list item applies to your research, read the appropriate section before selecting a response.

Materials & experimental systems

n/a	Involved in the study	
x		Antibodies
x		Eukaryotic cell lines
x		Palaeontology
x		Animals and other organisms
x		Human research participants
x		Clinical data

Methods

n/a	Involved in the study	
x		ChIP-seq
x		Flow cytometry
x		MRI-based neuroimaging
