# Supplementary material for: Bundle sheath suberisation is required for C4 photosynthesis in a Setaria viridis mutant
Source: Commun Biol. 2021 Feb 26;4:254. doi: 10.1038/s42003-021-01772-4 (PMC7910553; doi:10.1038/s42003-021-01772-4)
Supplement: Supplementary file 1 — Supplementary information [file 42003_2021_1772_MOESM1_ESM.pdf]

Reviewers' comments:

Reviewer #1 (Remarks to the Author):

The authors present a study describing the generation of a mutant line in the C4 model Setaria viridis that lacks suberisation of the bundle sheath cell wall. Although suberisation of the BS has long been thought of as a crucial anatomical component of the C4 pathway, this study represents the first demonstration that this is likely true, which is exciting. However, I feel in its current format the study lacks details and sufficient analyses.

For example, the evidence presented for the involvement of the ABCG transporter gene could be stronger. The "subsequent bioinformatics" is not described in enough detail. How can the authors be certain that this specific mutation is responsible for the phenotype? What of the other 3 SNPs in Supplementary Figure 1? What is the transporter predicted to do? Based on homology with other ABCG-type transporters, what might be the impact of the mutated amino acid residue? What is unique about this ABCG-type transporter compared to others?

Line 92 - "Expression analysis performed using existing maize, rice and S. viridis data showed that ABCG transporter gene is highly expressed in leaves of maize and setaria but not in rice (Supplementary Fig. 2) suggesting involvement of the ABCG transporter gene in C4 leaf development." Are ABCG-type transporter typically involved in leaf development? More details would help support this hypothesis. Why not try to complement the plant to see if a wild-type phenotype is restored?

Table 1: "CO_2 assimilation rate" – it is not clear what the parameters associated with this are? (measurements at max Ca?) Where are the typical estimates associated with ACi and A/PAR curves (e.g. Vcmax, Jmax, Amax)? (small modification suggestion: a separate column for "n" might better).

Line 124 and Fig. 3A – It is surprising that the assimilation rate of the phenotype remains considerably lower than the WT even under high CO_2 concentrations in the ACi curves, and is quite different from the predicted model in Supp Fig. 6. The apparent lack of a plateau in the ACi curve for the mutant is also worth discussing. As the authors suggest in the discussion, the mutant likely suffers from other "photosynthesis-affecting" complications (even though the authors show that PEPC, Rubisco and carbonic anhydrase activity levels were unchanged). No hypothesis is provided as to how the phenotype might be attributable to the ABCG-type transporter e.g. for the two hypotheses presented (1) the provision of PEP to PEP carboxylase must become limiting at higher fluxes (potentially via a limitation in ATP supply) 2) posttranslational downregulation of enzyme activity in the M is occurring)

Overall, the mutant is a good find and the physiological characterisations are interesting and sound, but I feel the author's should provide more details and/or work for the ABCG-type transporter here, or re-consider the presentation of the data and more moderately say that the observed phenotype was associated with the mutation but that further work is required to understand if/how it is responsible.

Reviewer #2 (Remarks to the Author):

Manuscript review

Manuscript#: COMMSBIO-20-0864-T
Bundle sheath suberisation is required for C4 photosynthesis in a <i>Setaria viridis</i> mutant By

Danila et al.

This manuscript describes the characterization of a <i>Setaria viridis</i> mutant affected in an ABCG transporter gene (<i>Sevir.9G451500.1</i>). Mutant plants showed a reduction in growth and photosynthesis compared to WT controls that the authors attribute to defective suberization of BS cell walls. BS suberization in C4 species is hypothesized to restrict exchange of gases and photosynthetic intermediates across the BS/M interface, but biochemical confirmation is currently lacking. Thus, the work makes an important contribution by providing genetic and biochemical evidence in support this long standing hypothesis.

Major concerns

1. How does this ABCG protein relate to other characterized half-transporters of this family? A phylogenetic three for ABCG genes for selected grass species (including maize, rice and <i>S. viridis</i>), Arabidopsis and potato (where ABCG transporters involved in suberization have been characterized) would be useful to put this new gene in context and determine if it is phylogenetically related to transporters involved in either cutin or suberin biosynthesis. This figure could be included as SI material.

2. The authors claim that the mutation of a ABCG gene leads to deficient suberization of the bundle sheath. Evidence provided for this is based on fluorescence microscopy after staining with Nile Red, TEM and GCMS analysis. However, I have the following observations/concerns:
2.1. A chemical analysis is needed to confirm the the "normal" suberization of the root endodermis. If suberization changes are small, microscopy with Nile Red would not detect such differences. However, small changes in endodermis suberization may still impact plant growth. Furthermore, what was the rationale for choosing Nile Red? Certainly, the fact that stains the intracellular space too makes the interpretation of the images confusing because not just suberin is stained.
2.2. The Nile Red staining of the mutant leaf cross section (Fig.2a) seems to indicate that both suberin and the cuticle are affected when compared to the image of the WT leaf section. The authors should discuss this observation. If the cuticle is also altered, this may have a large influence in the observed mutant phenotype.
2.3. Evidence presented by comparison of the TEM images shown are not totally convincing. It seems like a suberized layer is still present in the mutant (Fig.2b).
In terms of the chromatographic traces shown in Fig.2C, it is preferably to show graphs with the monomers identified and their quantities, typically as a histogram. The authors used a metabolomic approach to identify and quantify monomers based on their molecular ions. However, I could not access cited metabolome repository (MetabolomeExpress) to corroborate how this tool is used. Nevertheless, inspection of the metabolites listed on Table S1 as "suberin monomers" lead me to realize that specific suberin monomers are not really being analyzed here. Except for coumarate and ferulate (which may derive in part from suberin, but in grasses a large percentage may be released from lignified tissues) monomers listed are unsubstituted fatty acids of various lengths. Fatty acids are found in suberin but also in other cellular and extracellular lipids; thus, differences based solely on these compounds could not be unequivocally attributed to changes in suberin composition. None of the monomers that characterize suberin, namely ω-hydroxyacids, ω-hydroxy-epoxyacids and/or ɑ,ω-dicarboxylic acids are included. I suggest the authors identify suberin monomers by comparison to published mass spectra- in comparison to that of internal standards with known quantities.
2.4. Chemistry analyses used whole leaf samples (line 248). This means that cutin from the cuticle is also present in the leaf samples analyzed (i.e., the cuticle/epidermis was not separated from the leaf during sample preparation). Although not mentioned on the manuscript, the mix of monomers from both suberin and cutin has to be also considered in the interpretation of the data and discussed. Because Nile Red staining seems to show cuticle changes in the mutant (Fig.2a) and cuticular ledges

in the stomata (Fig.S4), it is possible that the cutin polyester is also affected. Therefore, a comparison of polyester monomer composition from whole leaves does not clearly indicate changes in suberin, cutin or both. Suberized bundle sheath-enriched tissues could be separately analyzed and compared following the protocol described in Spelie and Kolattukudy (1979) Composition of the aliphatic components of suberin from the bundle sheaths of <i>Zea Mays</i> leaves, Plant Science Letters 15, 225–230.

Taken together, from the arguments detailed above it is clear that a more thorough chemical characterization of suberized tissues of mutant and WT plants (using a validated method for suberin characterization) is needed to provide direct evidence supporting (or not) the conclusions drawn by the authors.

Other more specific comments.

Line 30. Replace "Main" with "Introduction".
Line 70. Provide a reference for the statement that this ABCG transporter is specifically expressed in C4 leaves.
Lines 105-106. Suberin has not been described as guard cell components; instead, the available literature refers to "cuticular ledges". Thus, this implies that cutin is also affected in this mutant.
Line 113. Provide a reference for the depolymerization method employed.
Lines 112-116. From the results shown here it cannot be concluded or suggested that remaining suberin is composed of the aromatic domain only.
Line 247. Provide a reference for the depolymerization method and GC/MS analysis described in this subsection.
Lines 255-257. Three internal standards were added (and this has a purpose); for better estimation of each compound quantity, it is important to use an IS of similar chemical characteristics as a reference. Usually, 1-pentadecanol is used to quantify fatty alcohols, 17:0 FAME to quantify fatty acid methyl esters and dicarboxylic fatty acids dimethyl esters, and 15:0 15-OH FAME to quantify hydroxy fatty acids. However, the authors seem to use only 1-pentadecanol to quantify compounds based on molecular ion counts (line 304). I find the chemical approach confusing.

Reviewer #3 (Remarks to the Author):

This manuscript details the characterization of a Setaria mutant line, selected from a chlorophyll fluorescence screen of a NMU-generated mutant population. The authors suggest that one of the four SNPs in this mutant is creating a suberin-deficiency in the leaves, due to a single AA change in the sequence of an ABCG transporter gene, which has previously been functionally implicated in the bundleh sheath suberization. I find the mutant fascinating and the paper presents some interesting results. I've provided my specific comments in order of importance below.

1.Why was only this mutation selected for further analysis and not the other three SNPs in the mutant line? It would also be informative to elaborate how this ABCG is involved in suberin biosynthesis.

2.While the authors have focused primarily on CO_2 assimilation, this seems a bit one-sided. Several additional functional roles have been hypothesized for bundle sheath suberization aside from reducing CCM leakiness. In particular, restriction of passive water loss from the vasculature, separation of

transpirational efflux and phloem loading and forcing exchange of photosynthetic intermediates through symplastic PD routes have all been suggested as alternative hypotheses.

3.I have some concerns about the various measurements used to assess BS leakiness and BS conductance. It seems like an infinite conductance of gm was used, i.e. any differences in gm will be lumped in the BS leakiness estimation. However, for the estimation of gbs, the conductance measured here is for diffusion pathway across the entirety of the mesophyll cells and bundle sheath/measophyll interface, not just for the latter. Hence, it seems to me that if the mesophyll cells were generally smaller in the mutant plants, this may shortened the diffusive pathway and also have increased the gbs using this method.

4.Replication rates of the anatomical measurements in Table 1 are hard to interpret, please specify if these are biological replicates or technical replicates. This may also impact the statistical analysis, since only t-tests were used to compare means of all pooled replicates (technical + biological). Also explain why the replication rate of some measurements was so variable and very low in some cases (n=2 for pit-field measurements).

5.There is clearly a difference in Nile Red labelling of the leaf cross-sections, but since this stain does not selectively label only suberin, it is not very conclusive and it seems from the TEM images that a clear SL is still visible in the mutant leaves? I also don’t find the Nile red stain differences very convincing for the guard cells, since the entire image is magenta and the intensity looks pretty much similar between the two inserts.

6.In the discussion of gbs vs leakiness differences, the argument that a doubling in gbs should correspond to a doubling in leakiness is flawed. An increase in gbs would impair the concentrating mechanisms, hence reducing Cbs in concert with the increase in gbs. This reduces the concentration gradient driving the leak flux, which strongly dampens the response of leakiness relative to gbs, without a need for reduced PEP carboxylation.

7.For the gas exchange measurements in Table 1, please reiterate the measurement conditions here to avoid confusion with the growth conditions (2% CO2). Considering that plants were grown at different conditions (ambient/2% CO2), It would also be good to specify which plants were used for which sub-panel in Fig.3 and what the measurement conditions were and which leaf was used for measurements?

Response to Reviewers Comments
COMMSBIO-20-0864-T

Reviewer #1 (Remarks to the Author):

The authors present a study describing the generation of a mutant line in the C4 model Setaria viridis that lacks suberisation of the bundle sheath cell wall. Although suberisation of the BS has long been thought of as a crucial anatomical component of the C4 pathway, this study represents the first demonstration that this is likely true, which is exciting. However, I feel in its current format the study lacks details and sufficient analyses.

1.1. For example, the evidence presented for the involvement of the ABCG transporter gene could be stronger. The “subsequent bioinformatics” is not described in enough detail. How can the authors be certain that this specific mutation is responsible for the phenotype? What of the other 3 SNPs in Supplementary Figure 1? What is the transporter predicted to do? Based on homology with other ABCG-type transporters, what might be the impact of the mutated amino acid residue? What is unique about this ABCG-type transporter compared to others?

We have now revised and expanded this section on bioinformatics by explaining the analysis systematically (Line 92-117) and adding supporting figures and tables. To characterise the inheritance of the mutant phenotype, a segregating backcrossed population was generated (BC1F2). From this population, 25% of the segregants showed a mutant phenotype (Supplementary Fig. 1) suggesting that a single gene or locus was involved. Mapping-by-sequencing analysis identified three candidate SNPs in chromosome 9 but only two are trait-associated, one of which is the causal gene, Sevir.9G451500 (Fig. 1b; Supplementary Fig. 2). Characterisation of Sevir.9G451500 revealed it to be an ABCG half-transporter gene (Supplementary Fig. 4). Protein sequence and structure analysis of ABCG protein family members reveal that the mutated amino acid (R552) involved in Sevir.9G451500 is highly conserved (Supplementary Table 1) and its location in the peptide sequence is associated with previously reported key component of the transmission interface that is essential for ABCG protein folding, ATP hydrolysis, and substrate binding and transport (Khunweeraphong, N., et al. (2017). "The structure of the human ABC transporter ABCG2 reveals a novel mechanism for drug extrusion." Scientific Reports 7(1): 13767).

1.2. Line 92 - “Expression analysis performed using existing maize, rice and S. viridis data showed that ABCG transporter gene is highly expressed in leaves of maize and setaria but not in rice (Supplementary Fig. 2) suggesting involvement of the ABCG transporter gene in C4 leaf development.” Are ABCG-type transporter typically involved in leaf development? More details would help support this hypothesis. Why not try to complement the plant to see if a wild-type phenotype is restored?

The suggestion of Sevir.9G451500 gene involvement in C4 leaf development is in line with the suberisation of the bundle sheath cells. In addition to the leaf expression analysis performed in maize, setaria and rice, we have now included a phylogenetic tree analysis showing Sevir.9G451500 is a direct ortholog of monocot and dicot genes previously reported to be involved in suberin transport (Supplementary Fig. 5; Line 118-124) to support this statement. Complementation of the mutation would require cell specific promoters or gene editing as ectopic expression of this transporter in all tissues is likely to have deleterious effects. The ecotype A10 background used for

this mutant screen is no longer commonly used for transformation due to low transformation efficiencies. We regard complementation as out of scope of the current study.

1.3. Table 1: “CO2 assimilation rate” – it is not clear what the parameters associated with this are? (measurements at max Ca?) Where are the typical estimates associated with ACi and A/PAR curves (e.g. Vcmax, Jmax, Amax)? (small modification suggestion: a separate column for “n” might better).

Vcmax, Jmax and Amax are C_3 photosynthesis parameters. A separate column for “n” has now been added.

1.4. Line 124 and Fig. 3A – It is surprising that the assimilation rate of the phenotype remains considerably lower than the WT even under high CO2 concentrations in the ACi curves, and is quite different from the predicted model in Supp Fig. 6. The apparent lack of a plateau in the ACi curve for the mutant is also worth discussing. As the authors suggest in the discussion, the mutant likely suffers from other “photosynthesis-affecting” complications (even though the authors show that PEPC, Rubisco and carbonic anhydrase activity levels were unchanged). No hypothesis is provided as to how the phenotype might be attributable to the ABCG-type transporter e.g. for the two hypotheses presented (1) the provision of PEP to PEP carboxylase must become limiting at higher fluxes (potentially via a limitation in ATP supply) 2) posttranslational downregulation of enzyme activity in the M is occurring)

Additional experimental data now show that the mutation in the ABCG transporter gene, Sevir.9G451500 caused leaf-specific suberin phenotype, which suggests that most of the differences in growth observed in mutant plants compared to the wildtype plants are due to reduced CO_2 assimilation rate attributed to the inefficiency of the CO_2 concentrating mechanism in mutant due to the lack of functional suberin lamellae in the BS cell wall. Whilst this is the primary effect of the mutation, a variety of regulatory processes may be occurring to mitigate this primary effect, matching BS and M biochemical fluxes and minimising CO_2 leakage. We have observed such responses in experiments with transgenic C_4 Flaveria and these papers are referenced in the text (Line 230-239).

Overall, the mutant is a good find and the physiological characterisations are interesting and sound, but I feel the author’s should provide more details and/or work for the ABCG-type transporter here, or re-consider the presentation of the data and more moderately say that the observed phenotype was associated with the mutation but that further work is required to understand if/how it is responsible.

Thank you for your constructive criticism. We hope that we have now addressed your concerns.

Reviewer #2 (Remarks to the Author):

Manuscript review

Manuscript#: COMMSBIO-20-0864-T
Bundle sheath suberisation is required for C4 photosynthesis in a *Setaria viridis* mutant By Danila et al.

This manuscript describes the characterization of a *Setaria viridis* mutant affected in an ABCG transporter gene (*Sevir.9G451500.1*). Mutant plants showed a reduction in growth and photosynthesis compared to WT controls that the authors attribute to defective suberization of BS cell walls. BS suberization in C4 species is hypothesized to restrict exchange of gases and photosynthetic intermediates across the BS/M interface, but biochemical confirmation is currently lacking. Thus, the work makes an important contribution by providing genetic and biochemical evidence in support this long standing hypothesis.

Major concerns

2.1. How does this ABCG protein relate to other characterized half-transporters of this family? A phylogenetic three for ABCG genes for selected grass species (including maize, rice and *S. viridis*), Arabidopsis and potato (where ABCG transporters involved in suberization have been characterized) would be useful to put this new gene in context and determine if it is phylogenetically related to transporters involved in either cutin or suberin biosynthesis. This figure could be included as SI material.

The phylogenetic relationship of the candidate gene with its orthologs, including previously characterised ABCG suberin precursor transporters, have now been included as suggested as Supplementary Figure 5.

2.2. The authors claim that the mutation of a ABCG gene leads to deficient suberization of the bundle sheath. Evidence provided for this is based on fluorescence microscopy after staining with Nile Red, TEM and GCMS analysis. However, I have the following observations/concerns:
2.2.1. A chemical analysis is needed to confirm the the "normal" suberization of the root endodermis. If suberization changes are small, microscopy with Nile Red would not detect such differences. However, small changes in endodermis suberization may still impact plant growth. Furthermore, what was the rationale for choosing Nile Red? Certainly, the fact that stains the intracellular space too makes the interpretation of the images confusing because not just suberin is stained.

Both staining and GC/MS analysis of suberin (aliphatic and aromatic monomers) in roots did not show significant difference between wildtype and mutant (Supplementary Figs. 6 and 7; Supplementary Table 2). In addition to using Nile Red, which stains aliphatic suberin residues, and Basic Fuchsin, which is a specific stain for lignin, we also performed Fluorol Yellow staining, which is a widely used suberin stain. Also, recovery of the mutant phenotype with high CO_2 is indicative of a photosynthetic not root-mediated basis for reduced growth.

2.2.2. The Nile Red staining of the mutant leaf cross section (Fig.2a) seems to indicate that both suberin and the cuticle are affected when compared to the image of the WT leaf section. The authors should discuss this observation. If the cuticle is also altered, this may have a large influence in the observed mutant phenotype.

We have repeated the Nile Red staining, along with Fluorol Yellow staining to check whether cutin is also affected in mutant leaf. Results showed presence of cutin staining in both wildtype and mutant leaf epidermis (Supplementary Fig. 6), suggesting cutin synthesis is not significantly reduced in these plants. Moreover, orthogonal sections of cleared leaf tissue after Nile Red staining only showed reduced fluorescence signal in the bundle sheaths (Fig. 2b).

2.2.3. Evidence presented by comparison of the TEM images shown are not totally convincing. It seems like a suberized layer is still present in the mutant (Fig.2b).

We have reworded our statement to clarify this and use “disrupted and thinner” in place of “absence” (Line 122-124).

2.3. In terms of the chromatographic traces shown in Fig.2C, it is preferably to show graphs with the monomers identified and their quantities, typically as a histogram. The authors used a metabolomic approach to identify and quantify monomers based on their molecular ions. However, I could not access cited metabolome repository (MetabolomeExpress) to corroborate how this tool is used. Nevertheless, inspection of the metabolites listed on Table S1 as “suberin monomers” lead me to realize that specific suberin monomers are not really being analyzed here. Except for coumarate and ferulate (which may derive in part from suberin, but in grasses a large percentage may be released from lignified tissues) monomers listed are unsubstituted fatty acids of various lengths. Fatty acids are found in suberin but also in other cellular and extracellular lipids; thus, differences based solely on these compounds could not be unequivocally attributed to changes in suberin composition. None of the monomers that characterize suberin, namely ω-hydroxyacids, ω-hydroxy-epoxyacids and/or α,ω-dicarboxylic acids are included. I suggest the authors identify suberin monomers by comparison to published mass spectra- in comparison to that of internal standards with known quantities.

We have redone the GC/MS and data analyses as suggested. We identified these monomers and their quantities are now shown as histogram in Figure 3 and also listed in Supplementary Table 2. Information on the identification and quantification of the monomers are discussed in the Methods section and also listed in Supplementary Table 3.

2.4. Chemistry analyses used whole leaf samples (line 248). This means that cutin from the cuticle is also present in the leaf samples analyzed (i.e., the cuticle/epidermis was not separated from the leaf during sample preparation). Although not mentioned on the manuscript, the mix of monomers from both suberin and cutin has to be also considered in the interpretation of the data and discussed. Because Nile Red staining seems to show cuticle changes in the mutant (Fig.2a) and cuticular ledges in the stomata (Fig.S4), it is possible that the cutin polyester is also affected. Therefore, a comparison of polyester monomer composition from whole leaves does not clearly indicate changes in suberin, cutin or both. Suberized bundle sheath-enriched tissues could be separately analyzed and compared following the protocol described in Spelie and Kolattukudy (1979) Composition of the aliphatic components of suberin from the bundle sheaths of *Zea Mays* leaves, Plant Science Letters 15, 225–230.

The GC/MS and data analyses now include results from mature leaf, mature root, and isolated bundle sheath strands as suggested (see Figure 3, Supplementary Tables 2 and 3). The bundle sheath strands were isolated following the protocol described in Ghannoum et al. 2005. Faster Rubisco is the key to superior nitrogen-use efficiency in NADP-malic enzyme relative to NAD-malic enzyme C4 grasses. Plant Physiology 137, 638-650. Suberin is clearly significantly reduced in the bundle sheath cell preparation.

Taken together, from the arguments detailed above it is clear that a more thorough chemical characterization of suberized tissues of mutant and WT plants (using a validated method for suberin

characterization) is needed to provide direct evidence supporting (or not) the conclusions drawn by the authors.

Thank you for your constructive criticism. We hope that we have now addressed your concerns.

Other more specific comments.

2.5. Line 30. Replace “Main” with “Introduction”.

Done as suggested.

2.6. Line 70. Provide a reference for the statement that this ABCG transporter is specifically expressed in C4 leaves.

Done as suggested.

2.7. Lines 105-106. Suberin has not been described as guard cell components; instead, the available literature refers to “cuticular ledges”. Thus, this implies that cutin is also affected in this mutant.

Cutin is not affected in the mutant.

2.8. Line 113. Provide a reference for the depolymerization method employed.

Reference now provided (Line 148-151).

2.9. Lines 112-116. From the results shown here it cannot be concluded or suggested that remaining suberin is composed of the aromatic domain only.

We have reworded this by saying that the disrupted suberin lamellae is a result of defects in the aliphatic suberin component as supported by our staining and new GC/MS data showing significant reduction in aliphatic suberin monomers in mutant bundle sheath (Line 128-161; Figures 2 and 3; Supplementary Figure 6; Supplementary Table 2).

2.10. Line 247. Provide a reference for the depolymerization method and GC/MS analysis described in this subsection.

References now provided in the Methods section (Line 302-303).

2.11. Lines 255-257. Three internal standards were added (and this has a purpose); for better estimation of each compound quantity, it is important to use an IS of similar chemical characteristics as a reference. Usually, 1-pentadecanol is used to quantify fatty alcohols, 17:0 FAME to quantify fatty acid methyl esters and dicarboxylic fatty acids dimethyl esters, and 15:0 15-OH FAME to quantify hydroxy fatty acids. However, the authors seem to use only 1-pentadecanol to quantify compounds based on molecular ion counts (line 304). I find the chemical approach confusing.

We have redone our GC/MS data analysis as suggested (Supplementary Tables 2 and 3).

Reviewer #3 (Remarks to the Author):

This manuscript details the characterization of a Setaria mutant line, selected from a chlorophyll fluorescence screen of a NMU-generated mutant population. The authors suggest that one of the four SNPs in this mutant is creating a suberin-deficiency in the leaves, due to a single AA change in the sequence of an ABCG transporter gene, which has previously been functionally implicated in the bundleh sheath suberization. I find the mutant fascinating and the paper presents some interesting results. I've provided my specific comments in order of importance below.

3.1. Why was only this mutation selected for further analysis and not the other three SNPs in the mutant line? It would also be informative to elaborate how this ABCG is involved in suberin biosynthesis.

This has been addressed above (see response to Reviewer 1 under 1.1).

3.2. While the authors have focused primarily on CO2 assimilation, this seems a bit one-sided. Several additional functional roles have been hypothesized for bundle sheath suberization aside from reducing CCM leakiness. In particular, restriction of passive water loss from the vasculature, separation of transpirational efflux and phloem loading and forcing exchange of photosynthetic intermediates through symplastic PD routes have all been suggested as alternative hypotheses.

Impaired water transport has been ruled out as a potential effect of the mutation because the mutant showed strong growth response to high CO_2 concentration, which is strongly indicative of CCM leakiness. We have also looked at functional effect on symplastic transport by comparative analysis of PD density but no significant change was observed between wildtype and mutant (Table 1; Supplementary Fig. 8).

3.3. I have some concerns about the various measurements used to assess BS leakiness and BS conductance. It seems like an infinite conductance of gm was used, i.e. any differences in gm will be lumped in the BS leakiness estimation. However, for the estimation of gbs, the conductance measured here is for diffusion pathway across the entirety of the mesophyll cells and bundle sheath/measophyll interface, not just for the latter. Hence, it seems to me that if the mesophyll cells were generally smaller in the mutant plants, this may shortened the diffusive pathway and also have increased the gbs using this method.

The gbs of mesophyll cells is in two order of magnitude smaller than the gbs of bundle sheath cells and therefore has negligible effect (See equation 3 in Alonso-Cantabrana, H., et al. (2018). Plant Physiology 178(1): 72-81)

3.4. Replication rates of the anatomical measurements in Table 1 are hard to interpret, please specify if these are biological replicates or technical replicates. This may also impact the statistical analysis, since only t-tests were used to compare means of all pooled replicates (technical + biological). Also explain why the replication rate of some measurements was so variable and very low in some cases (n=2 for pit-field measurements).

A separate column for "n" is now added in Table 1 to clarify this.

3.5. There is clearly a difference in Nile Red labelling of the leaf cross-sections, but since this stain

does not selectively label only suberin, it is not very conclusive and it seems from the TEM images that a clear SL is still visible in the mutant leaves? I also don't find the Nile red stain differences very convincing for the guard cells, since the entire image is magenta and the intensity looks pretty much similar between the two inserts.

These have been addressed above (see response to Reviewer 2 under 2.2.).

3.6. In the discussion of gbs vs leakiness differences, the argument that a doubling in gbs should correspond to a doubling in leakiness is flawed. An increase in gbs would impair the concentrating mechanisms, hence reducing Cbs in concert with the increase in gbs. This reduces the concentration gradient driving the leak flux, which strongly dampens the response of leakiness relative to gbs, without a need for reduced PEP carboxylation.

We have removed the phrase "not more than double as suggested by the determination of gbs" to avoid confusion.

3.7. For the gas exchange measurements in Table 1, please reiterate the measurement conditions here to avoid confusion with the growth conditions (2% CO2). Considering that plants were grown at different conditions (ambient/2% CO2), It would also be good to specify which plants were used for which sub-panel in Fig.3 and what the measurement conditions were and which leaf was used for measurements?

Done as suggested.

Reviewers' comments:

Reviewer #1 (Remarks to the Author):

Thanks to the authors for their comprehensive responses. I feel they have sufficiently dealt with the issues I raised. I did have one final query:

Based on the footnote in Table 1, CO2 assimilation rate appears to be derived from an A/PAR curve. I believe Asat values can be derived for C4 plants? Are these Asat values? If so it may be more accurate/clear to call this Asat or something more specifically associated with a max photo rate. Otherwise define the irradiance that this measurement is associated with.

Reviewer #2 (Remarks to the Author):

Bundle sheath suberisation is required for C4 photosynthesis in a Setaria viridis mutant
By Dasnila et al.

I thank the authors for taking my suggestions in consideration and undertaking the extra work to address my concerns. However, I have the following additional comments:

Microscopy
In their response letter, authors state: "We have repeated the Nile Red staining, along with Fluorol Yellow staining to check whether cutin is also affected in mutant leaf. Results showed presence of cutin staining in both wildtype and mutant leaf epidermis (Supplementary Fig. 6), suggesting cutin synthesis is not significantly reduced in these plants. Moreover, orthogonal sections of cleared leaf tissue after Nile Red staining only showed reduced fluorescence signal in the bundle sheaths (Fig. 2b)." To me, both Nile Red and Fluorol Yellow staining of the mutant leaf cross section (Supplementary Fig.6) do not clearly show that differences are limited to bundle sheath suberization; the cuticle seems similarly affected (i.e, fainter in the mutant than in the control sections). That said, I agree that the orthogonal view of z-stacks show decreased suberization in the bundle sheaths, which is also visible in the stained cross-sections.

GC/MS
The chemical analysis results shown in Figure 3 and in Supplementary Figure 7 are not clearly presented and the data dispersion is too large (particularly in Fig.S7) to make any meaningful conclusions; this method does not usually give such large error bars specially if the amount of tissue was ~0.25 g per replicate. It is unusual to report areas normalized to internal standard areas because that does not account for differences in tissue amounts used in each replicate. The authors are encouraged to re-plot their results by using internal standards to quantify each monomer and then normalize to the amount of tissue issued in each replicate (to get monomer mass per mass unit of tissue; such as ug/g). The description in the experimental section mentions 250 mg of tissue per replicate; was this high amount (1 g total) actually recovered from this laborious separation process? Were 250 mg exactly measured so that no tissue normalization is needed?
In lines 148-152 the authors state "GC/MS analysis of suberin monomers obtained after BF3-methanol depolymerisation of solvent-extracted residue from whole leaf and isolated BS strands revealed a significant reduction of up to 50% in the aliphatic suberin compounds (C16-C26) in mutant relative to wildtype plants (Fig. 3; Supplementary Table 2). No significant change was found between wildtype and mutant aromatic suberin monomers in leaf and isolated BS strands (Fig. 3; Supplementary Table 2)."
The results do not explain how aliphatics compare between the BS samples and the whole leaf

samples (only aromatic suberin monomers are compared). From what I see in Fig.3 and in the microscopy mentioned above, it is still unclear whether changes in aliphatic amounts are limited to changes in bundle sheath suberin alone.

The authors used the BF3-methanol depolymerization method to analyze lipid polyesters, but cite a paper (line 149) that uses a different catalyst (NaOMe), therefore it is unclear what is the reference for the protocol that was actually employed for the analysis.

Response to Reviewers Comments
COMMSBIO-20-0864A

We like to thank our reviewers for spending additional time to look at our revisions and for giving us further comments that we agree will make our paper better. Below is our point-by-point response that we hope addressed each concern. Changes made are highlighted in yellow within the manuscript text file.

Reviewer #1 (Remarks to the Author):

Thanks to the authors for their comprehensive responses. I feel they have sufficiently dealt with the issues I raised. I did have one final query:

Based on the footnote in Table 1, CO2 assimilation rate appears to be derived from an A/PAR curve. I believe Asat values can be derived for C4 plants? Are these Asat values? If so it may be more accurate/clear to call this Asat or something more specifically associated with a max photo rate. Otherwise define the irradiance that this measurement is associated with.

We do not like using the term A_{sat} as it is often not possible to measure light saturated CO_2 assimilation rates in C_4 plants. Table 1 footnote has now been revised to clearly define the irradiances used for the values listed under the gas exchange measurement parameters (Line 423-427).

Reviewer #2 (Remarks to the Author):

Bundle sheath suberisation is required for C4 photosynthesis in a Setaria viridis mutant
By Danila et al.

I thank the authors for taking my suggestions in consideration and undertaking the extra work to address my concerns. However, I have the following additional comments:

Microscopy
In their response letter, authors state: ”We have repeated the Nile Red staining, along with Fluorol Yellow staining to check whether cutin is also affected in mutant leaf. Results showed presence of cutin staining in both wildtype and mutant leaf epidermis (Supplementary Fig. 6), suggesting cutin synthesis is not significantly reduced in these plants. Moreover, orthogonal sections of cleared leaf tissue after Nile Red staining only showed reduced fluorescence signal in the bundle sheaths (Fig. 2b).” To me, both Nile Red and Fluorol Yellow staining of the mutant leaf cross section (Supplementary Fig.6) do not clearly show that differences are limited to bundle sheath suberization; the cuticle seems similarly affected (i.e, fainter in the mutant than in the control sections). That said, I agree that the orthogonal view of z-stacks show decreased suberization in the bundle sheaths, which is also visible in the stained cross-sections.

Electron micrographs of adaxial and abaxial cuticles of wildtype and mutant leaves show no significant difference between wildtype and mutant plants suggesting that cutin deposition in leaves of mutant plants is not affected by the mutation. This additional data is now included in the revised manuscript as Supplementary Figure 7.

GC/MS

The chemical analysis results shown in Figure 3 and in Supplementary Figure 7 are not clearly presented and the data dispersion is too large (particularly in Fig.S7) to make any meaningful conclusions; this method does not usually give such large error bars specially if the amount of tissue was ~0.25 g per replicate. It is unusual to report areas normalized to internal standard areas because that does not account for differences in tissue amounts used in each replicate. The authors are encouraged to re-plot their results by using internal standards to quantify each monomer and then normalize to the amount of tissue issued in each replicate (to get monomer mass per mass unit of tissue; such as ug/g). The description in the experimental section mentions 250 mg of tissue per replicate; was this high amount (1 g total) actually recovered from this laborious separation process? Were 250 mg exactly measured so that no tissue normalization is needed?

We have now replotted Figure 3 and Supplementary Figure 8 as suggested. Supplementary Table 2 was also updated to list values as relative concentration (μg monomer per g tissue) instead of peak area for each monomer. The description in the experimental section regarding the amount of tissue used per replicate was also clarified. We have now indicated that for both mature leaf and mature root samples, 250 mg of fresh tissues were collected per replicate while 2 mL or ~150 mg dried tissue per replicate was obtained for isolated bundle sheath strands (Line 306-309). For both mature leaf and mature root, 250 mg was used for normalisation but for bundle sheath strands, the exact tissue dry weight (after freeze-drying) was used.

In lines 148-152 the authors state "GC/MS analysis of suberin monomers obtained after BF3-methanol depolymerisation of solvent-extracted residue from whole leaf and isolated BS strands revealed a significant reduction of up to 50% in the aliphatic suberin compounds (C16-C26) in mutant relative to wildtype plants (Fig. 3; Supplementary Table 2). No significant change was found between wildtype and mutant aromatic suberin monomers in leaf and isolated BS strands (Fig. 3; Supplementary Table 2)."

The results do not explain how aliphatics compare between the BS samples and the whole leaf samples (only aromatic suberin monomers are compared). From what I see in Fig.3 and in the microscopy mentioned above, it is still unclear whether changes in aliphatic amounts are limited to changes in bundle sheath suberin alone.

Additional sentences were now added in the results section explaining how the reduction in the aliphatic compounds in mutant plants is attributed to BS suberin (Line 148-156). Replotting Figure 3 and addition of Supplementary Figure 7 greatly help in addressing this concern. Thank you.

The authors used the BF3-methanol depolymerization method to analyze lipid polyesters, but cite a paper (line 149) that uses a different catalyst (NaOMe), therefore it is unclear what is the reference for the protocol that was actually employed for the analysis.

To reference the BF_3-methanol depolymerisation step used, we have now cited Kolattukudy, P. E. & Agrawal, V. P. Structure and composition of aliphatic constituents of potato tuber skin (suberin). Lipids 9, 682-691, doi:10.1007/BF02532176 (1974), which specifically used BF_3-methanol for the depolymerisation step (Lines 149 and 313-316).

REVIEWERS' COMMENTS:

Reviewer #2 (Remarks to the Author):

All the points I have raised have been properly addressed and the modifications performed along the peer-review process have improved the manuscript. As I pointed out before, the role of BS suberization in C4 species is hypothesized to restrict exchange of gases and photosynthetic intermediates across the BS/M interface, but biochemical confirmation is currently lacking. This works makes an important contribution providing genetic and biochemical evidence in support of this long standing hypothesis.

I have a couple of very minor corrections:

Supplementary Table 2 and Supplementary Table 3:
"Methyl 18-oxidanyloctadeca-9,12-dienoate, TMS derivative" should be changed to: “Methyl 18-hydroxyoctadeca-9,12-dienoate, TMS derivative”

Supplementary Figure 8: not sure why the y axis scale goes up to 40 ug/g since the highest number is about 16 ug/g; please modify to make the graph clearer.
